# Fast and long-lasting immune response to S-trimer COVID-19 vaccine adjuvanted by PIKA

**DOI:** 10.1186/s43556-021-00054-z

**Published:** 2021-09-27

**Authors:** Yuan Liu, Lianpan Dai, Xiaoli Feng, Ran Gao, Nan Zhang, Bin Wang, Jianbao Han, Qingcui Zou, Xiling Guo, Hua Zhu, Jiangning Liu, Chuan Qin, Yi Zhang, Linlin Bao, Minghua Li

**Affiliations:** 1 YishengBio Co., Ltd, Beijing, China; 2 CAS Key Laboratory of Pathogenic Microbiology and Immunology, Institute of Microbiology, Chinese Academy of Sciences, Beijing, China; 3 Kunming National High-Level Biosafety Research Center for Non-Human Primates, Center for Biosafety Mega-Science, Kunming Institute of Zoology, Chinese Academy of Sciences, Kunming, China; 4 National Animal Models for Human Diseases Resources Center, NHC Key Laboratory of Human Disease Comparative Medicine, Beijing Key Laboratory for Animal Models of Emerging and Remerging Infectious Diseases, Institute of Laboratory Animal Science, Chinese Academy of Medical Sciences and Comparative Medicine Center, Peking Union Medical College, Beijing, China; 5 National Health Commission of the People’s Republic of China, Key Laboratory of Enteric Pathogenic Microbiology (Jiangsu Provincial Center for Disease Control and Prevention), Nanjing, China

**Keywords:** COVID-19, SARS-CoV-2, S-trimer, PIKA, Vaccine, Variants

## Abstract

In the face of the emerging variants of SARS-CoV-2, there is an urgent need to develop a vaccine that can induce fast, effective, long-lasting and broad protective immunity against SARS-CoV-2. Here, we developed a trimeric SARS-CoV-2 S protein vaccine candidate adjuvanted by PIKA, which can induce robust cellular and humoral immune responses. The results showed a high level of neutralizing antibodies induced by the vaccine was maintained for at least 400 days. In the study of non-human primates, PIKA adjuvanted S-trimer induced high SARS-CoV-2 neutralization titers and protected from virus replication in the lung following SARS-CoV-2 challenge. In addition, the long-term neutralizing antibody response induced by S-trimer vaccine adjuvanted by PIKA could neutralize multiple SARS-CoV-2 variants and there is no obvious different among the SARS- CoV-2 variants of interest or concern, including B.1.351, B.1.1.7, P.1, B.1.617.1 and B.1.617.2 variants. These data support the utility of S-trimer protein adjuvanted by PIKA as a potential vaccine candidate against SARS-CoV-2 infection.

**Supplementary Information:**

The online version contains supplementary material available at 10.1186/s43556-021-00054-z.

## Introduction

As of Sep 2021, according to the WHO, more than 221 million infections and nearly 4.6 million deaths were confirmed with coronavirus disease 2019 (COVID-19) worldwide [1]. Although great progress has been made in the development of COVID-19 vaccines, and several vaccines entering the clinical stage have been proved to induce protective immune response against severe acute respiratory syndrome coronavirus 2 (SARS-CoV-2), the persistence of humoral immune response induced by these vaccines is not clear, which is very important to ending this pandemic. In addition, the recent emergence of novel circulating variants has attracted great attention to the efficacy of these vaccines [2–6]. In fact, recently completed vaccine trials have shown that the overall efficacy is reduced in countries such as South Africa, Brazil and Israel, and there are significant geographical differences in the efficacy of mild and moderate diseases. In these countries, the epidemic is dominated by variant strains [4, 7, 8]. Therefore, there is an urgent need to develop interventions that can prevent the transmission of a variety of SARS-CoV-2 variants, including vaccine boosters for these variant strains or technologies that can stimulate widely neutralizing antibodies and long-term protective immune responses.

Up to now, most of COVID-19 vaccines have been developed through the use of spike protein as an antigen to produce a protective immune response to SARS-CoV-2 [9]. Spike protein is a major viral surface glycoprotein of SARS-CoV-2 that interacts with human angiotensin-converting enzyme 2 (hACE2). More and more evidence showed that compared with monomer antigen, multimerized antigen interacts better with B cell receptor, so as to promote the production of high affinity antibody. Some studies have shown that several modified proteins (such as RBD dimer and S-trimer) can induce higher levels of neutralizing antibodies than monomer proteins [10].

In addition to the design of immunogens, adjuvants also play a significant role in inducing long-lasting and robust antibody response. Currently, only a few adjuvants have been approved for use in humans, including alum, AS01, AS03, AS04, MF59, and CpG 1018. Some promising adjuvants are currently undergoing human safety and immunogenicity tests [11–13]. PIKA adjuvant, as a synthetic chemical analogue of a double-stranded RNA (dsRNA), acts similarly as other dsRNA in activating an immune response through recognition by TLR3 [14–16]. Another important dsRNA-signaling pathway is initiated by the RNA helicase RIG-I. Sequential elevation of TNF-α, IL-6, IL-12p40 and IFN-γ is evident in mice serum after intramuscular administration of PIKA [17]. It also promotes the maturation and activation of dendritic cells and up-regulate co-stimulatory molecules, such as CD86, CD80, HLA-DR, CD83, and HLA class-I on dendritic cell. The non-specific activation of innate immunity by PIKA adjuvant could therefore create an ideal environment for antigens to be presented in generating specific immunity. Recent phase I and phase II clinical studies of inactivated purified rabies virus adjuvanted by PIKA (PIKA Rabies vaccine) showed that the vaccine was safe and more immunogenic or non-inferior immunogenicity than the commercially available vaccine in healthy adults [18, 19]. These encouraging study data strongly support PIKA as an adjuvant of COVID-19 vaccine.

Here, we studied the immunogenicity and protective efficacy of S-trimer protein adjuvanted by PIKA in rabbits, mice and nonhuman primates. Rabbit studies showed that the S-trimer protein adjuvanted with PIKA induced fast, high, and long-lasting neutralizing antibody against SARS-CoV-2 virus. Adjuvanting the S-trimer with PIKA induced potent cellular immune response and showed complete protection from SARS-CoV-2 infection in mice. Furthermore, studies in nonhuman primates demonstrated that S-trimer adjuvanted by PIKA induced high neutralization titer and protected from virus replication in the lung following SARS-CoV-2 virus challenge. In addition, the long-term neutralizing antibody response induced by S-trimer vaccine adjuvanted by PIKA could neutralize multiple SARS-CoV-2 variants and there is no obvious difference among the SARS- CoV-2 variants of interest (VOI) or concern (VOC), including B.1.351, B.1.1.7, P.1, B.1.617.1 and B.1.617.2 lineage viruses. These results support the S-trimer protein adjuvanted by PIKA as a potential COVID-19 vaccine candidate.

## Results

### S-trimer adjuvanted with PIKA induces fast, robust, long-lasting and broad neutralizing antibody response against SARS-CoV-2 in rabbits

**Fig. 1:**
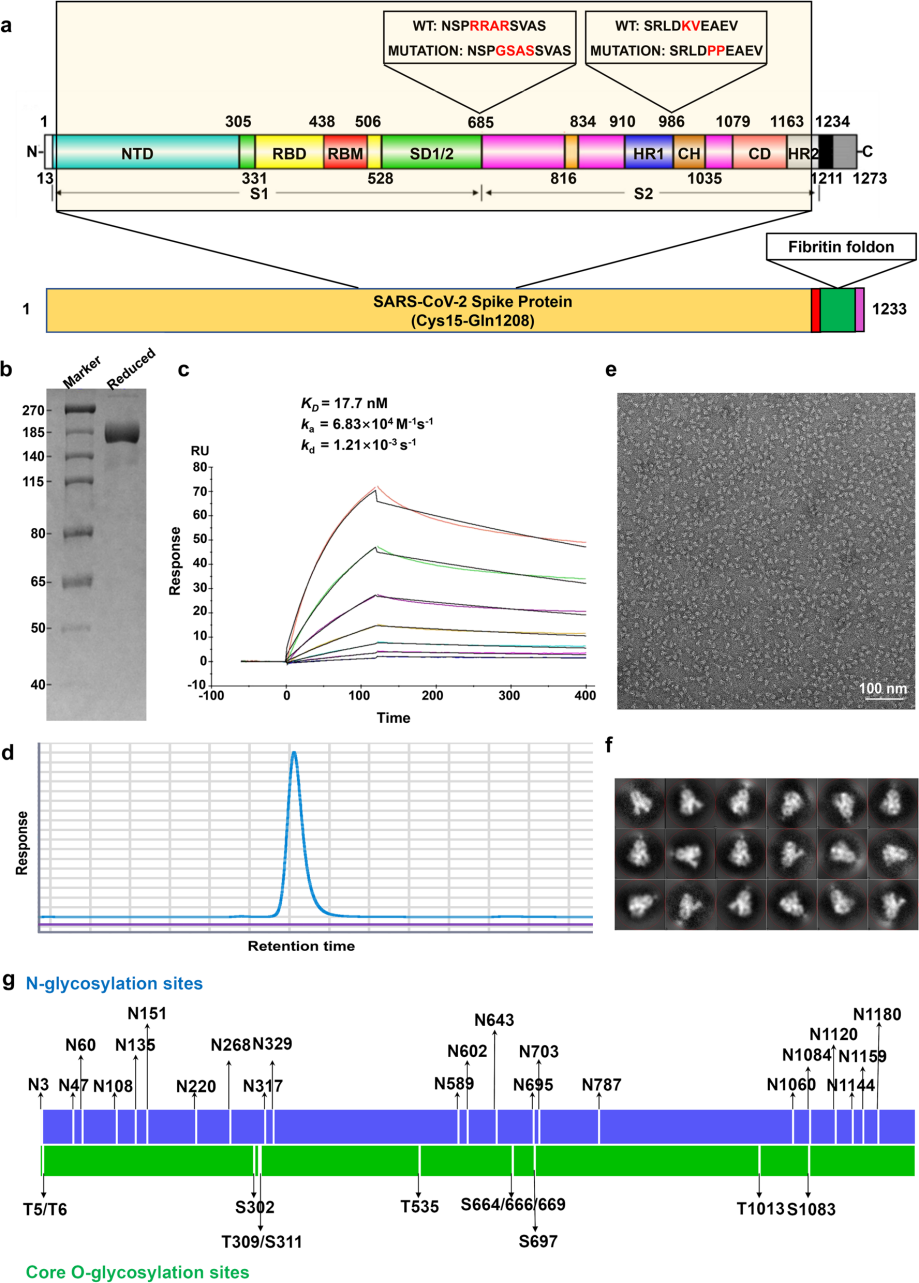
Characterization of the SARS-CoV-2 S-trimer. **a** Schematic design of the S-trimer. **b** Reducing SDS-PAGE analysis of the S-trimer. **c** The real-time binding profile between theS-trimer protein and ACE2 characterized using surface plasmon resonance (Biacore). **d** SEC-HPLC analysis of the purity of the S-trimer. **e** Negative staining TEM image of the S-trimer. **f** 2D class-averaged images of the SARS-CoV-2 S-trimer obtained by negative staining TEM. **g** Glycosylation profile on the S-trimer characterized by nanoLC-MS/MS

To express a trimeric form of prefusion stabilized full-length S protein, we fused the trimerization domain of T4 to the amino acid residues 15–1208 of SARS-CoV-2 spike protein (Fig. 1a). After stable transfection, monoclonal CHO cells with high titer were screened by the expression of target protein. After process optimization, a fed batch serum-free cell culture process in bioreactor was developed. Recombinant S-trimer was successfully purified and the purity of the S-trimer was more than 95% (Fig. 1d). SDS-PAGE result showed the apparent molecular mass of the monomer of S-trimer protein was around 170 kDa (Fig. 1b), which was larger than the molecular mass calculated using the monomer of S-trimer amino acid sequence alone (about 137 kDa). The larger molecular mass may be related to the high glycosylation of the S-trimer. Through high resolution mass spectrometry, we identified 22 N- glycosylation sites and 9 core O-glycosylation sites (Fig. 1g).

To determine whether the trimer is folded correctly, we measured the binding activity of the trimer to human ACE2 using surface plasmon resonance(SPR) (Fig. 1c). We also observed negatively stained S-trimer protein using TEM. Figure 1e showed the uniform triangle structures. The two-dimensional class averaged image of S-trimer protein resulted in a clearer image shown in Fig. 1f, showing different open and closed states of RBD.

**Fig. 2:**
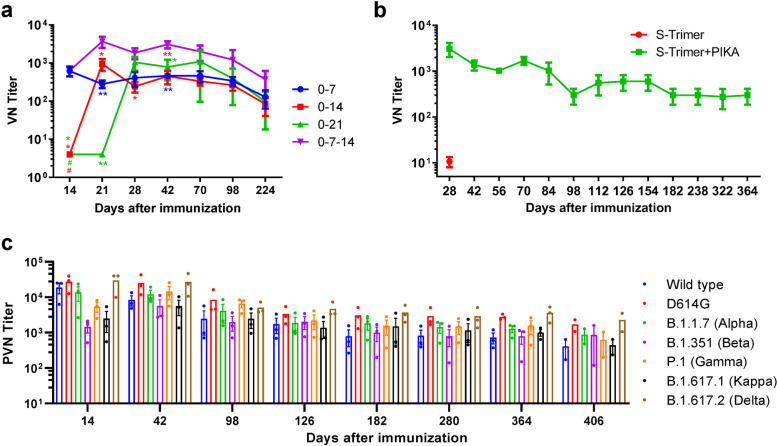
Humoral Immune Response of the S-trimer Vaccine adjuvanted by PIKA in Rabbits. **a** Rabbit neutralization antibody levels with different immunization regimens. Rabbits were injected intramuscularly with the S-trimer Vaccine adjuvanted by PIKA by twice immunization (D0/D21; D0/D14; D0/D7) or by three times immunization (D0/D7/D14) and neutralizing antibody levels were detected using wild-type SARS-CoV-2. Data are shown as mean ± SEM. *P*-values were analyzed with a two-way ANOVA (vs. 0–7-14 group, *p < 0.05, **p < 0.01; vs. 0–7 group, #*p* < 0.05). **b** Rabbits were injected intramuscularly with the S-trimer Vaccine adjuvanted by PIKA at 0, 7 and 14 days, and neutralization antibody levels at 28, 42, 56, 70, 84, 98, 112, 126, 154, 182, 238, 322, 364 and 406 days after the first dose were determined by using wild-type SARS-CoV-2. **c** Rabbits were injected intramuscularly with the S-trimer Vaccine adjuvanted by PIKA at 0, 7 and 14 days, and pseudovirus neutralization antibody levels at 14, 42, 98, 126, 182, 280, 364 and 406 days after the first immunization were determined using the recombinant VSV-based SARS-CoV-2 pseudovirus

The results of antigen comparison study (Supplementary Fig. S1) and adjuvant comparison study (Supplementary Fig. S2) showed that the immunogenicity of S-trimer was higher than that of S1 Protein and RBD protein, and the adjuvant activity of PIKA adjuvant was better than that of other adjuvants. To further assess the immunogenicity of S-trimer adjuvanted with PIKA, rabbits were intramuscularly immunized with variant regimen of PIKA-adjuvanted S-trimer. For two-dose regimen, we evaluated different immunization regimens (D0/D21, D0/D14 and D0/D7 intervals) which rabbits were immunized at days 0/21, days 0/14, and days 0/7, respectively (Fig. 2a). No significant differences were found among these three groups during the whole observation period (from 28 to 224 days). Moreover, the immunized rabbits developed neutralizing antibody responses only after the second immunization, reaching similar level of neutralizing antibodies in the groups that immunized with different immunization interval programs. It implies that in the case of COVID-19's sudden outbreak, earlier booster shots could benefit to give protection to the vaccinated population earlier. We also evaluated the antibody response of a three-dose regimen, immunizing mice at day 0, day 7, and day 14. The results showed that during the entire observation period, the three-dose (D0/D7/D14) regimen resulted in a higher level of neutralizing antibodies than the two-dose regimen (Fig. 2a). One week after last immunization, the NT50 titers in rabbits immunized with three-dose or two-dose approached ~ 1/3,500 and ~ 1/900, respectively. Importantly, the high neutralization antibody level has been sustained for 224 days so far. To further confirm the long-term immune response, we observed the level of neutralizing antibody by the microcytopathogenic effect assay method using infectious SARS-CoV-2 for 1 year. The results showed that the neutralization potency of the serum gradually decreased with the extension of observation time and remained stable for 364 days from ~ 3000 to ~ 300 (Fig. 2b).

To test the potential neutralizing response against new variants induced by S-trimer adjuvanted with PIKA, sera from three rabbits, receiving three doses at days 0, 7, 14 were obtained on certain days after prime immunization. We assayed the neutralization activity against SARS-CoV-2 pseudotyped viruses including the spike protein of the wild-type strain, and several circulating variants, including D614G, B.1.351 (Beta), B.1.1.7 (Alpha), P.1 (Gamma), B.1.617.1 (Kappa) and B.1.617.2 (Delta). Our studies showed that all serum samples collected starting from Day 14 (two dose inoculation completed) post the prime immunization were able to neutralize the above five types of pseudoviruses. Compared with neutralizing antibody response against the wild-type strain (geometric mean titer [GMT] 15734), post-vaccination sera (Day 14) were similarly effective in neutralizing D614G (GMT 24534), B.1.1.7 (GMT 9750) and B.1.617.2 (GMT 25106) variants, whereas serum neutralization potency decreased against B.1.351 (GMT 1235), P.1 (GMT 4844) and B.1.617.1 (GMT 1653) (Fig. 2c). Consistent with recent reports using serum samples from mRNA vaccine or inactivated viral vaccine recipients [5, 20], we demonstrated that several VOCs (e.g. B.1.1.7) are effectively neutralized although there is RBD mutations, whereas VOCs with E484Q or E484K mutations showed resistance to serum neutralization of immune individuals in the early stage of immunization. For long-term humoral immunity, we evaluated the neutralization potency of the serum samples collected from Day 42 to Day 406 against above six types of pseudoviruses (Fig. 2c). Along with the time passed by post vaccination, the neutralization potency of the serum decreased gradually and finally reached stable level from Day 182 day to Day 406, which was in line with the results of live SARS-CoV-2 virus. More interestingly, with time prolonged, the difference between different variants disappeared (Fig. 2c), reflecting long-term humoral immunity induced by S-trimer adjuvanted with PIKA could neutralize multiple SARS-CoV-2 variants.

The results of further expanding the sample size also verify the results (Supplementary Fig. S3a). Eight rabbits were immunized intramuscularly twice (Days 0 and 7) with S-trimer adjuvated with PIKA. We assayed the serum samples from the 14- and 84-day time points respectively. Similarly, the short-term (Day14) neutralization activity of serum against the above five types of pseudoviruses was significantly different, but there was no appreciable difference for long-term (Day 84) neutralization activity. Consistently, compared with those specific to wildtype RBD, the titers of IgG specific to E484Q containing RBD were significantly reduced at Day 14, which could contribute to immune escape by VOCs containing the mutations at residue positions 484. However, there was no significant difference in the IgG of serum on day 84 (Supplementary Fig. S3b).

### S-trimer adjuvanted with PIKA induces a balanced Th1/Th2 immune response in mice

**Fig. 3:**
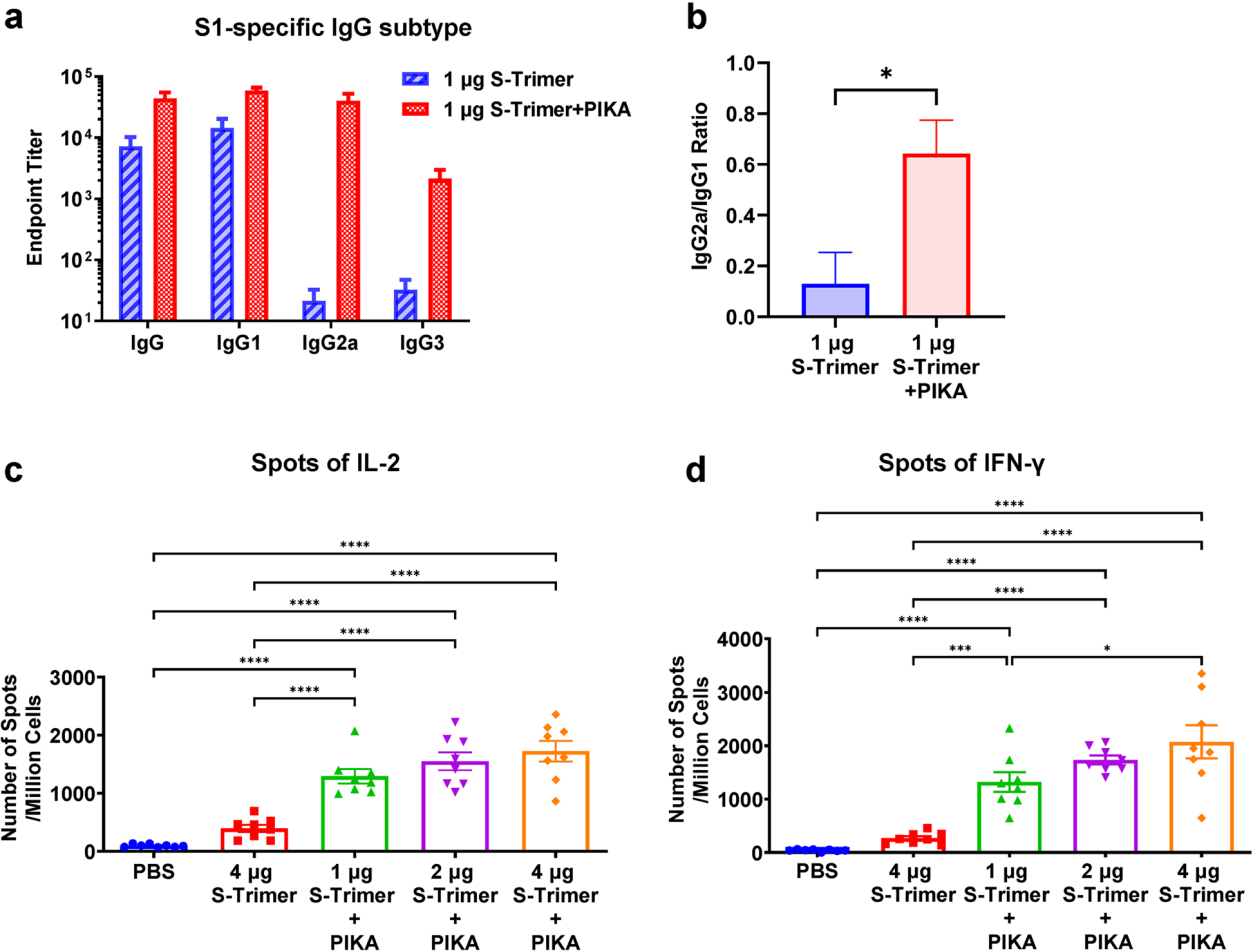
Immunogenicity of the S-trimer Vaccine adjuvanted by PIKA in mice. **a** and **b** BALB/c mice (*n* = 7–8/group) were immunized with 1 μg S-trimer with or without PIKA adjuvant three times on Day 0, Day 7 and Day 14. The humoral immune responses on Day 35 were analyzed based on S1 protein binding antibody ELISA titers. Titers of IgG and subytpes of IgG including IgG1, IgG2a and IgG3 produced and the ratio of IgG2a/IgG1 were determined. Data are shown as mean ± SEM. *P*-values were analyzed with unpaired t- test (*p < 0.05). **c** and **d** C57BL/6 J mice (*n* = 8/group) were immunized with various doses of S-trimer with or without PIKA adjuvant twice on Day 0 and Day 7. At 7 days after last injection, splenocytes were harvested from mice and re-stimulated with peptide pool for SARS-CoV-2 S protein (2 μg/ml of each peptide), followed by detection of IL-2 and IFN-γ cytokines by ELISpot. Points represent individual animals. Data are shown as mean ± SEM. *P*-values were analyzed with a one-way ANOVA (*p < 0.05, **p < 0.01, ***p < 0.001, ****p < 0.0001)

We further evaluated the IgG subclass response of mice immunized with S-trimer adjuvanted with or without PIKA to determine Th1/Th2 polarization. S-trimer adjuvanted with PIKA raised not only IgG1 responses but also IgG2a, while S-trimer only induced IgG1 responses (Fig. 3a&b). High titers of the IgG1 antibody reflect Th2 immunity, while high titers of the IgG2a antibody isotype denote Th1 immunity. This indicated a shift in immunity towards balanced Th1/Th2 response.

We also studied the cellular immune response of SARS-CoV-2 S-trimer protein in mice. Splenocytes were collected 14 days after the last immunization and re-stimulated in vitro with SARS-CoV-2 S protein peptide pool (Fig. 3c&d). We observed that the S-trimer adjuvanted with PIKA significantly upregulated the frequency of IL-2 and IFN-γ secreting T helper cells. Interestingly, in groups immunized with S-trimer adjuvanted with PIKA, the inductions of IFN-γ and IL-2 was three times or higher than that of animals immunizing S-trimer alone, suggesting that PIKA adjuvant may help to initiate T-cell immune response.

The study of SARS-CoV-2-specific T cells stimulated by ex vivo antigen stimulation after the third immunization also showed T cell response in the S-trimer vaccine adjuvanted with PIKA groups. In contrast, mice receiving the S-trimer alone cannot developed T-cell responses (Supplementary Fig. S4). S-specific CD8 + T cells producing IFN-γ, IL-2, TNF-α or IL-4 were measured in all animals immunizing the S-trimer adjuvanted by PIKA, with particularly high IL-2, TNF-α and IL-4-secreting CD8 + T-cell levels (Fig. S4). In addition, Th1 cytokines IL-2, TNF- α and IFN-γ were significantly enhanced in the high dose group of PIKA adjuvanted S-trimer. However, there was no obvious change in the frequency of S-specific CD4 + T cells producing Th2 cytokine (IL-4) in the vaccine group compared with the control group (Supplementary Fig. S4).

### S-trimer adjuvanted with PIKA induces rapid cellular immune response in mice

**Fig. 4:**
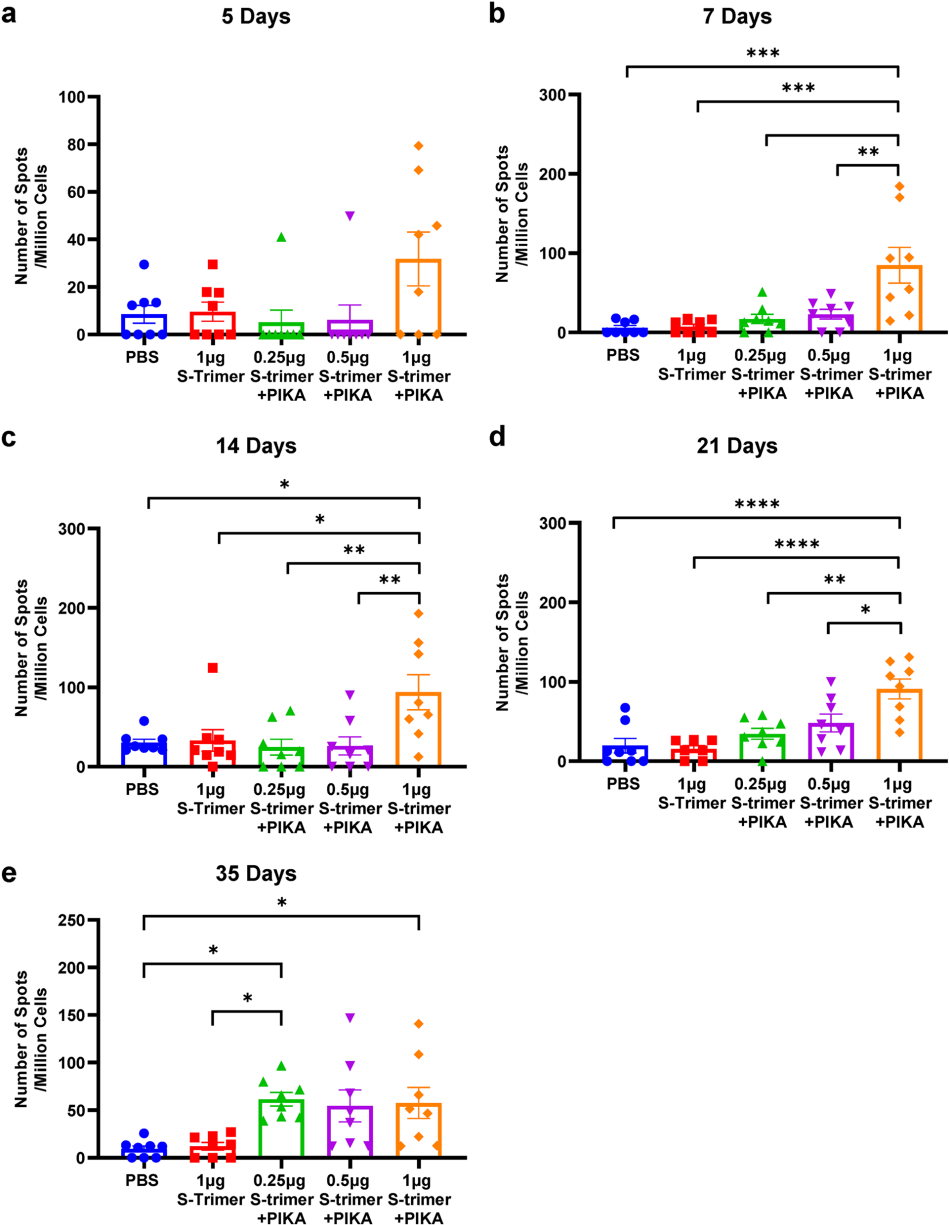
Time Course of Cellular Immune Response of the S-trimer Vaccine adjuvanted by PIKA in mice. BALB/c mice were immunized with different doses of S-Trimer with or without PIKA adjuvant three times on Day 0, Day 7 and Day 14. Splenocytes were harvested from mice (*n* = 8/group) at 5, 7, 14, 21 and 35 days after the first dose and re-stimulated with the S-trimer protein (4 μg/ml), followed by detection of IFN-γ cytokines by ELISpot. Points represent individual animals. Data are shown as mean ± SEM. *P*-values were analyzed with a one-way ANOVA (*p < 0.05, **p < 0.01, ***p < 0.001, ****p < 0.0001)

Then we tested whether the ability of PIKA adjuvant to promote immune activation facilitates a rapid cellular immune response after immunization. Mice were administrated with different doses of S-trimer with or without PIKA adjuvant three times on Day 0, Day 7 and Day 14. Splenocytes were collected from 5 to 35 days after the first dose and re-stimulated with the S-trimer protein ex-vivo, followed by detection of IFN-γ cytokines by ELISpot (Fig. 4a-e). A rapid enhancement of frequency of IFN-γ secreting T cells was observed 5 days post immunization in the high-dose of S-trimer adjuvanted with PIKA. The frequency gradually increased in all S-trimer adjuvanted with PIKA groups as the time prolonged. In contrast, animals immunized with S-trimer alone had absent induction of IFN-γ.

### S-trimer adjuvanted with PIKA provides protection from SARS-CoV-2 replication and infection in the lungs of mice and nonhuman primates

**Fig. 5:**
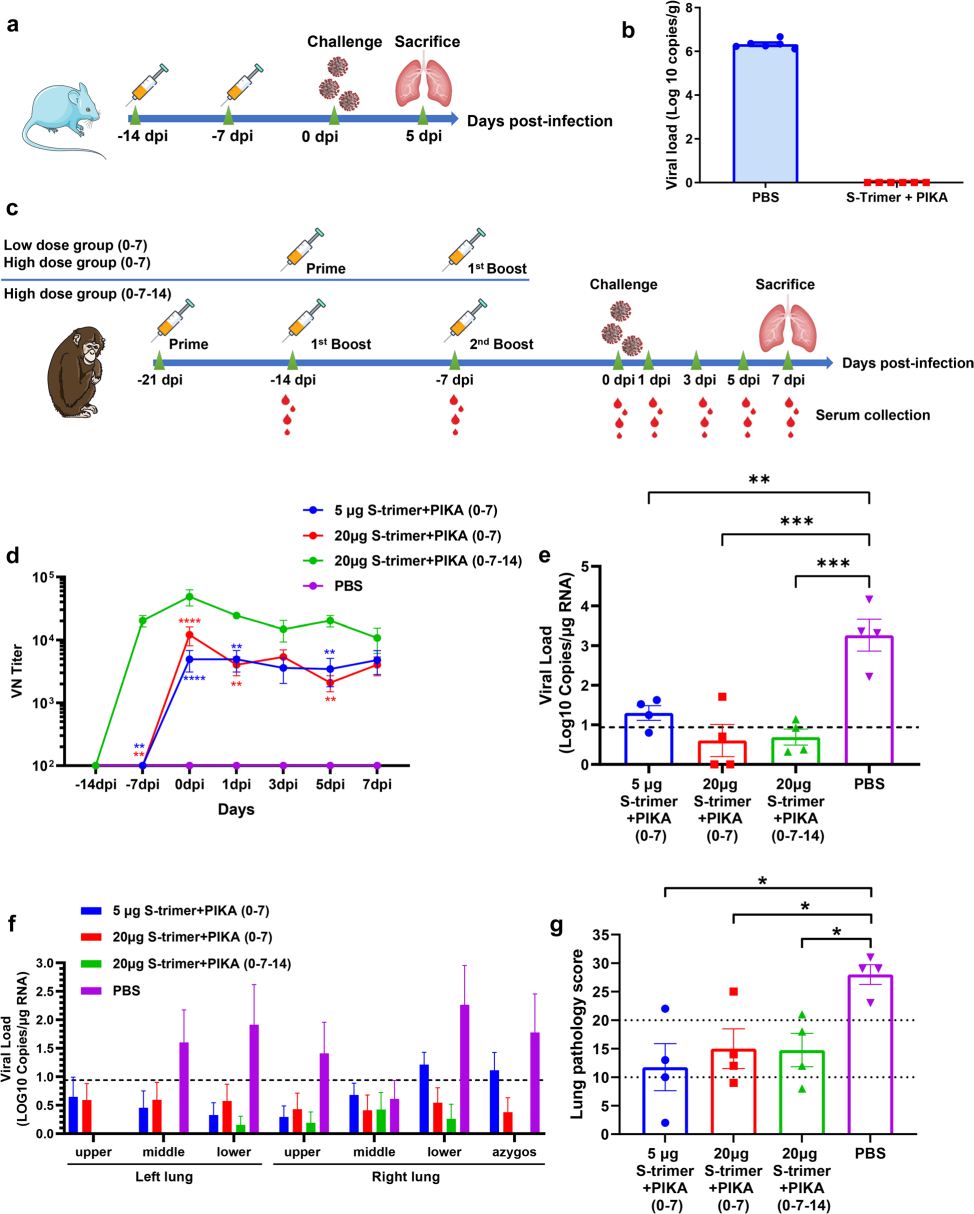
Protective Efficacy of the S-trimer adjuvanted by PIKA in hACE2 transgenic mice and Nonhuman Primates. **a** Flow chart of the study design in hACE2 transgenic mice. Groups of 6-week-old hACE2 transgenic mice (*n* = 6) were vaccinated with two doses of 1 μg S-trimer with PIKA adjuvant in one-week intervals. PBS were given as controls. At 7 days after last immunization, mice were challenged with 50 TCID_50_ of SARS-CoV-2 intranasally. Lung tissues were harvested 5 days after challenge. **b** Viral RNA load in the lung was detected by qRT-PCR. Data are shown as mean ± SEM. **c** Flow chart of the study design in nonhuman primates. Groups of 5–7 years old cynomolgus (*n* = 4) were vaccinated with two or three doses of 5 μg or 20 μg S-trimer with PIKA adjuvant in one-week intervals. PBS were given as controls. At 7 days after last immunization, cynomolgus were challenged with 1 × 10^7^ TCID_50_ of SARS-CoV-2 (40% intranasal and 60% intratracheal). Lung tissues were harvested 7 days after challenge. Serum was collected after initial vaccination for antibody assays. **d** Neutralizing antibody levels were detected using wild-type SARS-CoV-2. Data are shown as mean ± SEM. *P*-values were analyzed with a two-way ANOVA (*vs* 20 μg S-trimer + PIKA (0–7-14) group, **p < 0.01, ****p < 0.0001). **e** and **f** Viral RNA load in the lung was determined by qRT-PCR. Data are shown as mean ± SEM. The horizontal dashed line indicates the limit of detection. Significance was calculated using one-way ANOVA (**p < 0.01; ***p < 0.001). **g** Score of lung pathology at Day 7 post-infection. See Table S1 for additional details. Data are shown as mean ± SEM. *P*-values were analyzed with unpaired t- test (*p < 0.05)

To evaluate the efficacy of S-trimer adjuvanted with PIKA, we challenged vaccinated hACE2 transgenic mice with SARS-CoV-2 intranasally at 7 days after the last immunization. Following challenge, we detected the viral RNA to measure the virus load in the lung at 5 days post inoculation (Fig. 5a). The results showed that S-trimer protein adjuvated with PIKA provided protection from SARS-CoV-2 infection. In the S-trimer adjuvanted with PIKA immunization group, the viral load in lung tissue was completely lower than the detection limit, but a higher level of viral load was detected in PBS group (Fig. 5b).

The immunogenicity of S-trimer adjuvanted with PIKA was further evaluated in nonhuman primates (cynomolgus) (Fig. 5c). Cynomolgus (*n* = 4 per group) were immunized with two or three doses of 5 μg or 20 μg S-trimer with PIKA adjuvant in one-week intervals. PBS were given as controls. The animals were then challenged at 7 days after last immunization, with 1 × 10^7^ TCID_50_ (40% intranasal and 60% intratracheal) SARS-CoV-2 virus. High levels of neutralizing antibodies against wild-type SARS-CoV-2 virus were found in all vaccinated animals, while no neutralizing antibodies were detected in the control group (Fig. 5d). In agreement with the rabbit results, the neutralization antibody level induced by three-dose programme was significantly higher than induced by two-dose programme. There were no significant differences in the neutralization antibody titer between 5 μg and 20 μg S-trimer groups vaccinated with two doses.

Next, we evaluated the protective effect of S-trimer adjuvanted with PIKA against SARS-CoV-2 infection in cynomolgus. All cynomolgus were euthanized 7 days after infection (DPI) to evaluate viral load and respiratory tissue damage. In line with the high neutralizing antibody levels, all cynomolgus vaccinated with 5 or 20 μg of S-trimer protein adjuvanted with PIKA showed protection against SARS-CoV-2 infection, and lower levels of viral RNA was found in the lung tissues (Fig. 5e&f), whereas higher levels of viral RNA were detected in the PBS group.

To evaluate lung pathology, multiple regions of the upper, middle and lower lung lobes were analyzed. Pathologists performed lung pathological analysis and scoring by blind method. In line with the results of virus load, the lung pathology score of vaccinated group was also lower than that of control group (Fig. 5g, Supplementary Table S1).

## Discussion

Since the first description of human infection with SARS-CoV-2 in December 2019, nine vaccines have been approved for human use. Nevertheless, the recent emergence of new variants has aroused great concern about the efficacy of these vaccines. In order to control the spread of SARS-CoV-2, an effective vaccine inducing rapid, long-lasting and broad protective immunity is urgently needed. Herein, we developed a COVID-19 vaccine based on S-trimer protein and PIKA adjuvant, and tested its immunogenicity and efficacy in rabbits, mice and non-human primates using PIKA adjuvant. Our study showed that CHO derived S-trimer protein with PIKA as adjuvant could trigger a strong immune response, indicating that CHO cells are a suitable platform for the production of S-trimer protein in vaccine development. So far, we have constructed stable CHO cell clones expressing S-trimer protein, and selected the clones with the high yield to produce the main cell bank and working cell bank for GMP production of commercial vaccines. In this study, we showed that the immunized animals can produce high NAb levels (EC50 titer of 1000) on two weeks after the first immunization by twice immunization (D0/D7), indicating that it may be suitable for emergency vaccination in high-risk populations. To study the durability of immune response, our observation period spanned more than 1 year. The results demonstrated that the neutralizing antibody could still be detected 1 year after immunization, no matter taking two- or three-times immunization. 406 days after the first immunization, the pseudovirus neutralizing antibody level gradually decreased from 10^4^ to 10^3^, indicating that the vaccine can achieve long-term humoral protection for no less than 13 months.

PIKA adjuvant is a class of synthesized double strand RNA (dsRNA) molecules. Endosomal dsRNA can be recognized by TLR3 while cytosolic dsRNA can be sensed by the retinoic acid-inducible gene I-like receptor family which include RIG-I and melanoma differentiation associated protein-5 (MDA-5) [21, 22]. TLR3 is expressed primarily endosomal and in multiple cell and tissue types, including muscle cells, epithelial cells, certain neoplasms and antigen presenting cells; the RIG-I and MDA-5 are ubiquitously expressed. Through TLR3, MDA-5 and RIG-I signal pathway, PIKA can induce a prompt production of interferon, cytokines, chemokines and co-stimulatory factors. The anti-viral and anti-tumor effects of interferon have been well established and led to FDA approval of several interferon-based products for antiviral and anti-tumor indications. In recent years, the U.S. FDA has approved several TLR adjuvanted vaccines, including TLR4 based HPV vaccine (Cerarix) and zoster vaccine (Shingrix), and TLR9 based HBV vaccine (Heplisav). Double-strand RNA stimulation can activate dendritic cells and upregulate the co-stimulatory and activation markers of dendritic cells such as CD86 and CD40 [17].

Protein-based vaccine without proper adjuvant is poorly presented by dendritic cells to CD8 T cells which are important for anti-viral effect. The production of type I interferon upon PIKA stimulation facilitates antigen cross-presentation by dendritic cells and augment CD8 T-cell and NK-cell responses, which makes protein-based vaccines suitable for viral clearance [17]. DsRNA is also found to activate NK cells through TLR-TICAm-1 pathway, and decrease both myeloid-derived suppressor cells and regulatory T cells, which also provide rationale for integrating PIKA in anti-viral treatment. Our results showed that S-trimer adjuvanted by PIKA can significantly enhanced the cellular immune response, which is supposed to be the better antiviral immune response to prevent vaccine-associated enhanced respiratory disease.

Many studies have shown that the level of neutralizing antibodies in vaccine recipients will gradually decrease over time [23]. The SARS-CoV-2 variants may further reduce the neutralization antibody activity. Recently, Moderna also reported the persistence of neutralizing antibody titers in quarterly reports in the first quarter [24]. The data showed that 6 to 8 months after the two doses of mRNA-1273, although most of the volunteers' sera still had neutralization ability to the wild-type virus, nearly half of the volunteers' sera had neutralization titers to beta (b.1.351) and gamma (P.1) below the detection level. The collective data on the mutation effect of variant strains on the efficacy of vaccine and convalescent serum show that the polyclonal antibody response is focused on several immune dominant regions, indicating that there is a high possibility of escaping host immunity through mutation in the future. With the emergence of different antigenic variants, it is essential to develop a vaccine that can induce stronger and long-lasting neutralization activity and wider neutralization breadth. Our study found that long-term humoral immunity induced by S-trimer adjuvanted with PIKA could neutralize multiple SARS-CoV-2 variants and there is no significant different between the variants. Two reasons may account for this result. First, the antibodies against conserved regions are retained and the antibodies against non-conserved regions targeting RBD are removed from the repertoire over time. Second, the evolution of antibody libraries has led to the acquisition of neutralization breadth over time. Studies have shown that the memory B cells of individuals infected with SARS-CoV-2 will appear clonal renewal after 6.2 months. Their antibodies have stronger anti-RBD mutation efficacy, indicating the continuous evolution of humoral response[25]. Persistent antibody evolution occurs in germinal centers, requiring B cells to be exposed to antigens captured in the form of immune complexes on follicular dendritic cells [25, 26]. S-trimer with PIKA as adjuvant may stimulate virus specific B cells for a long time. The epitopes targeted by the mutation-resistant IgG antibodies will be more and more presented to B cells through antibody feedback over time. This needs further investigation and verification.

In summary, we report a COVID-19 vaccine candidate with recombinant S-trimer protein as antigen and PIKA as adjuvant and provided evidence of immunogenicity and efficacy in multiple animal models. These results were supporting clinical trials of the vaccine candidate especially considering long-lasting and broad-spectrum of neutralizing antibodies. The safety, immunogenicity, and efficacy need to be investigated in clinical trials.

## Materials and methods

### Design, expression and purification of S-trimer

The S-trimer constructs contain the mammalian codon-optimized gene encoding the spike protein residues 15–1208 (SARS-CoV-2, GenBank: MN908947), followed by a thrombin cleavage site, a C-terminal T4 fibritin trimerization domain, and 6x-His tag subcloned into the expression vector PKS001. In order to prevent the cleavage of S1-S2 site, two amino acid site mutations were designed at S1-S2 cleavage site (RRAR to GSAS), and to stabilize the protein in the pre-fusion state, and two proline mutations were introduced in the HR1 domain (K986P and V987P). The plasmids were transfected into CHO cells and grown in serum-free medium. Then the high yield clones were screened, and a fed batch serum-free cell culture process in 50L bioreactor was developed. S-trimer protein was purified by immobilized metal ion affinity chromatography, low pH virus inactivation, anion exchange chromatography and hydrophobic charge induction chromatography. Finally, after nanofiltration and UF / DF steps, the S-trimer protein were obtained.

### PIKA adjuvant

PIKA is produced according to Good Manufacturing Practices by Liaoning Yisheng Biopharma Co., Ltd.

### Vaccine formulation

The purified recombinant S-trimer proteins at the different concentrations, were mixed with PIKA adjuvant. The formulations were prepared with concentrations of 2.5–20 μg/ml for S-trimer protein and 1 mg/ml for the PIKA adjuvant.

### Immunogenicity analysis of S-trimer in mice

Balb/c mice were randomly divided into five groups (40 mice in each group). On day 0, 7, and 14, mice in vaccine group were immunized intramuscularly (i.m.) with vaccine candidate at three doses (0.25 μg S-Trimer/0.1 ml, 0.5 μg S-Trimer/0.1 ml, 1 μg S-Trimer/0.1 ml). Mice in antigen control group were inoculated with antigen control vaccine, and mice in PBS control group were inoculated with PBS. Blood samples (0.5 ml each) were collected from eight mice in each group on day 35 after the initial immunization and tested for titers of S1-specific IgG and subtypes of IgG by ELISA. Splenocytes were harvested from mice on day 5, 7, 14, 21, and 35 after first immunization and stimulated with the S-trimer protein (4 μg/ml), followed by detection of IFN-γ cytokines by ELISpot and intracellular cytokine staining by flow cytometry.

C57BL/6 J mice were randomly divided into 5 groups (*n* = 8/group). On day 0 and 7, mice in vaccine group were immunized intramuscularly with PIKA S-trimer vaccine at three doses (1 μg S-trimer, 2 μg S-trimer and 4 μg S-trimer). At 7 days after last administration, splenocytes were harvested and re-stimulated with peptide pool for SARS-CoV-2 S protein (2 μg/ml of each peptide), followed by detection of IFN-γ and IL-2 cytokines by ELISpot.

### Immunogenicity analysis of S-trimer in rabbits

For the immune persistence test, New Zealand rabbits (*n* = 4/group) were immunized intramuscularly with the S-trimer adjuvanted by PIKA three times on Day 0, Day 7, and Day 14. Serum were collected from each rabbit in each group on day 28, 42, 56, 70, 84, 98, 112, 126, 154, 182, 238, 322, 364 and 406 after the initial immunization and tested for wild-type SARS-CoV-2 neutralizing antibodies to evaluate the immune persistence.

For the immune regimen exploration test, New Zealand rabbits (*n* = 5/group) were immunized with 5 μg S-trimer with or without PIKA adjuvant by twice immunization (Day 0 and Day 21; Day 0 and Day 14; Day 0 and Day 7) or by three times immunization (Day 0, Day 7, and D14). Serum were collected from each rabbit on day 14, 21, 28, 42, 70, 98, 140 and 224 after the initial immunization and tested for wild-type SARS-CoV-2 neutralizing antibodies to compare different vaccination schedule.

### Wild-type SARS-CoV-2 neutralization assay

Wild-type SARS-CoV-2 neutralization assays were performed at Jiangsu Provincial Center for Disease Control and Prevention (Nanjing, China). The activity of neutralizing antibodies was verified by microneutralization (MN) assay based on cytopathic changes. Serum samples were inactivated at 56 °C for 30 min. The diluted samples were mixed with a virus suspension of 100 TCID_50_, followed by 2 h incubation in a 5% CO_2_ incubator. Vero cells were then added to the serum-virus mixture, and the plates were incubated for 3–5 days in a 5% CO_2_ incubator. The neutralizing antibody titer was calculated by the dilution number of 50% protective condition.

### Pseudo-virus neutralization assay

SARS-CoV-2 (Wild-type, D614G, B.1.351, B.1.1.7, P1, B.1.617) pseudo-virus neutralization assays were performed at Gobond Testing Technology (Beijing) Co., Ltd. (Beijing, China). Vero cells were cultured in DMEM supplemented with 10% heat inactivated fetal bovine serum, 50 U/ml Penicillin–streptomycin solution at 37℃ with 5% CO_2_. Inactivated serum samples were serially dilute and incubated with 1.3 × 10^4^ TCID_50_/ml SARS-CoV-2 pseudo-typed virus for 1 h at 37 °C. Vero cells were added after 1 h and allowed to incubate for 24 h. Positive and negative control samples were prepared as same way. Post infection, cells were lysed and RLU were measured using the Microplate Luminometer. Neutralization titers were calculated as the serum dilution at which RLU were reduced by 50% compared with RLU in virus control wells.

### ELISA assay

Serum were collected from the animals after vaccination. ELISA plates were coated with recombinant RBD, E484Q RBD or S1 protein (Sino Biological Inc., Beijing, China) in the coating buffer at 4 °C overnight. Following blocking and incubation with serial dilutions of sera, anti-mouse IgG, IgG1, IgG2a or IgG3 HRP-conjugated antibody were used as secondary Abs and incubated for 1 h at RT. The TMB was used as the substrate to detect Ab responses. After reaction stopping, plates were read at 450 nm wavelength.

### Splenocyte stimulation and ELISpot assay

Splenocyte were collected and re-stimulated with S peptide pools at a final concentration of 2 μg/ml or purified recombinant S-trimmer protein at a final concentration of 4 μg/ml or 10 μg/ml on PVDF plates coated with anti-mouse IFN-γ and IL-2 antibody. The PVDF plates were incubated for 28 h. Following stimulation, cells were washed out with PBS prior to adding anti-mouse IL-2, and IFN-γ detection antibody solution and followed with 2 h RT incubation. The PVDF plates were washed with PBS, and Streptavidin-ALP solution was then added into plates and incubated for 1 h at RT. After washing, the plates were added BCIP/NBT solution and incubated for 30 min, then the plates were wash with purified water and dried at RT. Spots were scanned and quantified and Spot-forming unit (SFU) per million cells was calculated.

### SARS-CoV-2 viral challenge study in hACE2 transgenic mice

Experiments of hACE2 transgenic mice were performed in Institute of Laboratory Animal Sciences, CAMS & PUMC (Beijing, China). Groups of 6-week-old hACE2 transgenic mice (*n* = 6) were vaccinated with two doses of 1 μg S-trimer with PIKA adjuvant at Day 0 and Day 7. PBS were given as controls. At 7 days after last immunization, mice were challenged with 10^5^ TCID_50_/mouse of SARS-CoV-2 intranasally. Lung tissues were harvested 5 days after challenge and split for virus load detection and pathological examination.

### SARS-CoV-2 viral challenge study in non-human primates

Non-human primates challenge studies were performed in the Biosafety Level 3 (BSL-3) in the Kunming Institute of Zoology, CAS. Male cynomolgus (*n* = 12, age range 5 to 7 years) were divided randomly into 4 groups (low-dose, high-dose 1, high-dose 2, and placebo group). Animals in low-dose group were intramuscularly immunized with 5 μg S-Trimer. Animals in high-dose group were intramuscularly immunized with 20 μg S-Trimer. Animals in high-dose 2 and placebo group were administrated at day 0, 7, and 14 and injected. Animals in low-dose and high-dose 1 group were administrated at day 7 and 14. The viral challenge was conducted 7 days after the final injection by direct inoculation of 10^7^ TCID_50_ of SARS-CoV-2 virus (40% intranasal and 60% intratracheal). Neutralizing antibody test were conduct on day 7 after each immunization and day 1, 3, 5, and 7 after challenge. All cynomolgus were euthanized at 7 days after challenge. The viral RNA load in seven lobes was determined by qRT-PCR, and a pathological examination was conducted.

### Statistical analysis

Statistical analysis was performed using Prism 8.0 (GraphPad software). Multiple groups across multiple time points were compared using two-way ANOVA and Tukey’s multiple comparison post hoc tests. Comparisons among multiple groups were performed using one-way ANOVA, followed by Tukey's multiple comparison post hoc tests. The two groups were compared using an unpaired Student *t*-test. *P* values < 0.05 is considered to be significant. * *p* < 0.05, **p < 0.01, *** *p* < 0.001, ****p < 0.0001. NS, not significant.

## Supplementary Information

**Additional file 1: Figure S1.** Neutralizing antibodies induced by S-trimer, S1 and RBD protein with or without PIKA adjuvant in rabbits. **Figure S2.** Neutralizing antibodies induced by S-trimer adjuvanted by various adjuvants in rabbits. **Figure S3.** Pseudovirus Neutralizing antibodies and Anti-RBD IgG antibodies induced by S-trimer adjuvanted by PIKA in rabbits. **Figure S4.** SARS-CoV-2-Specific T Cell Immune Response of PIKA Adjuvanted S-trimer in Mice. **Supplementary Table 1.** Lung pathology scores post infection.

## Data Availability

The datasets generated during and/or analysed during the current study are available from the corresponding author on reasonable request.
