# Supplementary material for: Fast and long-lasting immune response to S-trimer COVID-19 vaccine adjuvanted by PIKA
Source: Mol Biomed. 2021 Sep 27;2:29. doi: 10.1186/s43556-021-00054-z (PMC8475395; doi:10.1186/s43556-021-00054-z)
Supplement: Supplementary file 1 — Additional file 1: Figure S1. Neutralizing antibodies induced by S-trimer, S1 and RBD protein with or without PIKA adjuvant in rabbits. Figure S2. Neutralizing antibodies induced by S-trimer adjuvanted by various adjuvants in rabbits. Figure S3. Pseudovirus Neutralizing antibodies and Anti-RBD IgG antibodies induced by S-trimer adjuvanted by PIKA in rabbits. Figure S4. SARS-CoV-2-Specific T Cell Immune Response of PIKA Adjuvanted S-trimer in Mice. Supplementary Table 1. Lung pathology scores post infection. [file 43556_2021_54_MOESM1_ESM.docx]

Fast and Long-lasting Immune Response to S-trimer COVID-19 Vaccine Adjuvanted by PIKA

**Supplemental Materials**

# Materials and methods

## SEC-HPLC

The purity of S-trimer was analyzed by Size-Exclusion Chromatography (SEC-HPLC) using Agilent 1260 Infinity HPLC with an analytic TSK gel UltraSW Aggregate column (Tosoh). The system was operated with a flow rate of 0.5 mL/min and a pressure of 120 bar. A total of 100 µL from each sample was added to the column (oven temperature 25 °C) and the measurement was carried out for 35 min.

## Receptor binding studies of S-Trimer to human ACE2

The surface plasmon resonance (SPR) assay was performed on a Biacore T200 (Biacore). Fc -tagged human ACE2 (desalted in a buffer containing 1×HEPES (10 mM HEPES, 150 mM NaCl, 3 mM EDTA) with 0.005%Tween-20 prior to Biacore analysis) was captured on an Anti-human IgG(Fc) antibody coated CM5 chip. S-trimer protein was injected at a speed of 30 μl/min and passed over the surface of the chip to an equivalent around 130 resonance units (RU). Resonance signals were recorded and analyzed by the Biacore T200 software to derive the equilibrium dissociation constant (KD).

## Negative staining electron microscopy

The morphologies of the S-trimer protein were characterized by bio-TEM (FEI Talos L120C) at an acceleration voltage of 120 kV.  Image processing and 2D class averaging were performed with cisTEM.

## Liquid chromatography–MS/MS analysis to identify glycosylation sites

To identify the glycosylation sites, we used the ultrasensitive Q-Exactive nanoLC-MS/MS system. The analysis was performed by National Engineering Research Center for Protein Drugs (Beijing, China).

### N-Glycosylation Site Analysis

The S-trimer protein were ultra-filtrated using 5mmol/L pH 7.0 phosphate buffer, then adding 8mol/L carbamide (final concentration 6mol/L), denaturizing for 1h at 4℃, followed by ultrafiltration using deionized water. Then the samples were treated by three enzymatic hydrolysis schemes: 1. DTT reduction and IAM alkylation after enzymatic hydrolysis of Chymotrypsin; 2. Enzymatic hydrolysis with Trypsin + Glu-c followed by DTT reduction and IAM alkylation; 3. Alpha-lytic Protease (aLP) enzymatic hydrolysis after DTT reduction and IAM alkylation.

The digestion mixture was separated on a nanoflow LC (nanoLC UltiMate3000) using a nano–electrospray ionization spray column (analytical column, C18, φ75 μm × 150 mm, 3 μm) with a gradient of 6–95% buffer B (100% acetonitrile and 0.1% formic acid) at a flow rate of 600 nl/min over 90 min, coupled on-line to a Q-Exactive mass spectrometer (Thermo Fisher Scientific) equipped with a nanospray ion source. The resulting MS/MS data were searched against an in-house database, using MASCOT (Matrix Science, Boston, MA).

### O-Glycosylation Site Analysis

The S-trimer protein were ultra-filtrated using 5mmol/L pH 7.0 phosphate buffer, then adding 8mol/L carbamide (final concentration 6mol/L), denaturizing for 1h at 4℃, followed by ultrafiltration using deionized water. The the samples were treated by two enzymatic hydrolysis schemes: 1. After enzymatic hydrolysis with Trypsin+Glu-C, N-sugar was cut by glycopeptide, then DTT reduction, and IAM alkylation; 2. Alpha-lytic Protease (aLP) enzymatic hydrolysis after DTT reduction and IAM alkylation, and N-sugar was cut.

The digestion mixture was separated on a nanoflow LC (nanoLC UltiMate3000) using a nano–electrospray ionization spray column (analytical column, C18, φ75 μm × 150 mm, 3 μm) with a gradient of 6–95% buffer B (100% acetonitrile and 0.1% formic acid) at a flow rate of 600 nl/min over 90 min, coupled on-line to a Q-Exactive mass spectrometer (Thermo Fisher Scientific) equipped with a nanospray ion source. The resulting MS/MS data were searched against an in-house database and MASCOT database, using MASCOT (Matrix Science, Boston, MA).

## Immunogenicity analysis of S-Trimer in rabbits

For the antigen screening test, New Zealand rabbits (n=4/group) were injected intramuscularly with the same dose (6μg/dose) of different recombinant proteins, including S-trimer, S1 and RBD, adjuvanted by PIKA adjuvant by three times immunization (Day 0, Day 7, and D14). Blood samples (0.5ml each) were collected from each rabbit in each group on day 28 after the initial immunization and tested for wild-type SARS-CoV-2 neutralization antibodies to compare different antigen.

For the adjuvant screening test, New Zealand rabbits (n=5/group) were injected intramuscularly with the same dose (5μg/dose) of S-trimer with PIKA, Alum, AddaS03, Quil-A or CpG adjuvant by twice immunization (Day 0 and Day 7). Blood samples (0.5ml each) were collected from each rabbit on day 14 after the initial immunization and tested for wild-type SARS-CoV-2 neutralization antibodies to compare different adjuvant.

## Flow cytometry

Intracellular cytokine staining was performed on splenocytes harvested from BALB/c mice stimulated with the purified recombinant S-trimmer protein. Cells were stained with the following antibodies: PE-Cy7 anti-mouse CD4, APC anti-mouse CD8a, APC-Cy7 anti-mouse CD3e, BV421 anti-mouse IL-4, BV510 anti-mouse CD4, BV711 anti-mouse CD8a, FITC anti-mouse IFN-γ, PE anti-mouse IL-2, PE-Cy7 anti-mouse CD69, APC anti-mouse TNF-α, and 7-AAD Dye. Cell were washed with flow dyeing buffer (PBS supplemented with 0.2% BSA). Cell events were acquired using an LSRFortessa flow cytometer followed by FlowJo software (FlowJo LLC, Ashland, OR) analysis.

**Supplemental Figures**


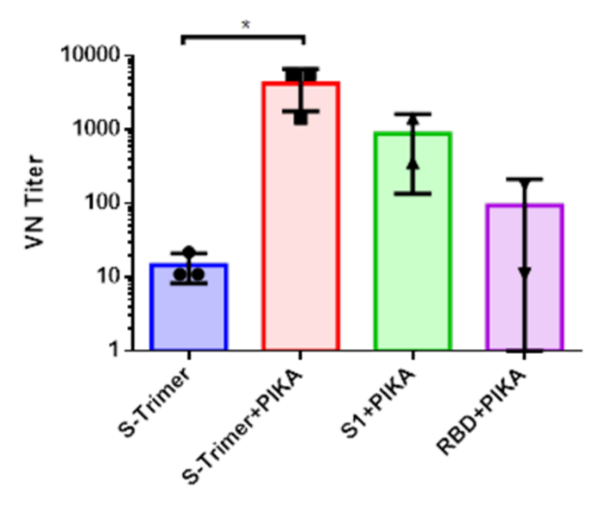


**Figure S1. Neutralizing antibodies induced by S-trimer, S1 and RBD protein with or without PIKA adjuvant in rabbits.** Rabbits were injected intramuscularly with the same dose (6μg/dose) of different recombinant proteins, including S-trimer, S1 and RBD, adjuvanted by PIKA by three times immunization (D0/D7/D14) and neutralization antibody levels were determined using infectious SARS-CoV-2 at 14 days after last immunization. Data are shown as mean ± SEM. Significance was calculated using a one-way ANOVA with multiple comparisons tests (**p* < 0.05).

**Figure S2. Neutralizing antibodies induced by S-trimer adjuvanted by various adjuvants in rabbits.** Rabbits were injected intramuscularly with the same dose (5μg/dose) of S-trimer with PIKA, Alum, AddaS03, Quil-A or CpG adjuvant by twice immunization (D0/D7) and neutralization antibody levels were determined using infectious SARS-CoV-2 at 7 days after last immunization. Data are shown as mean ± SEM. Significance was calculated using a one-way ANOVA with multiple comparisons tests (****p* < 0.001).


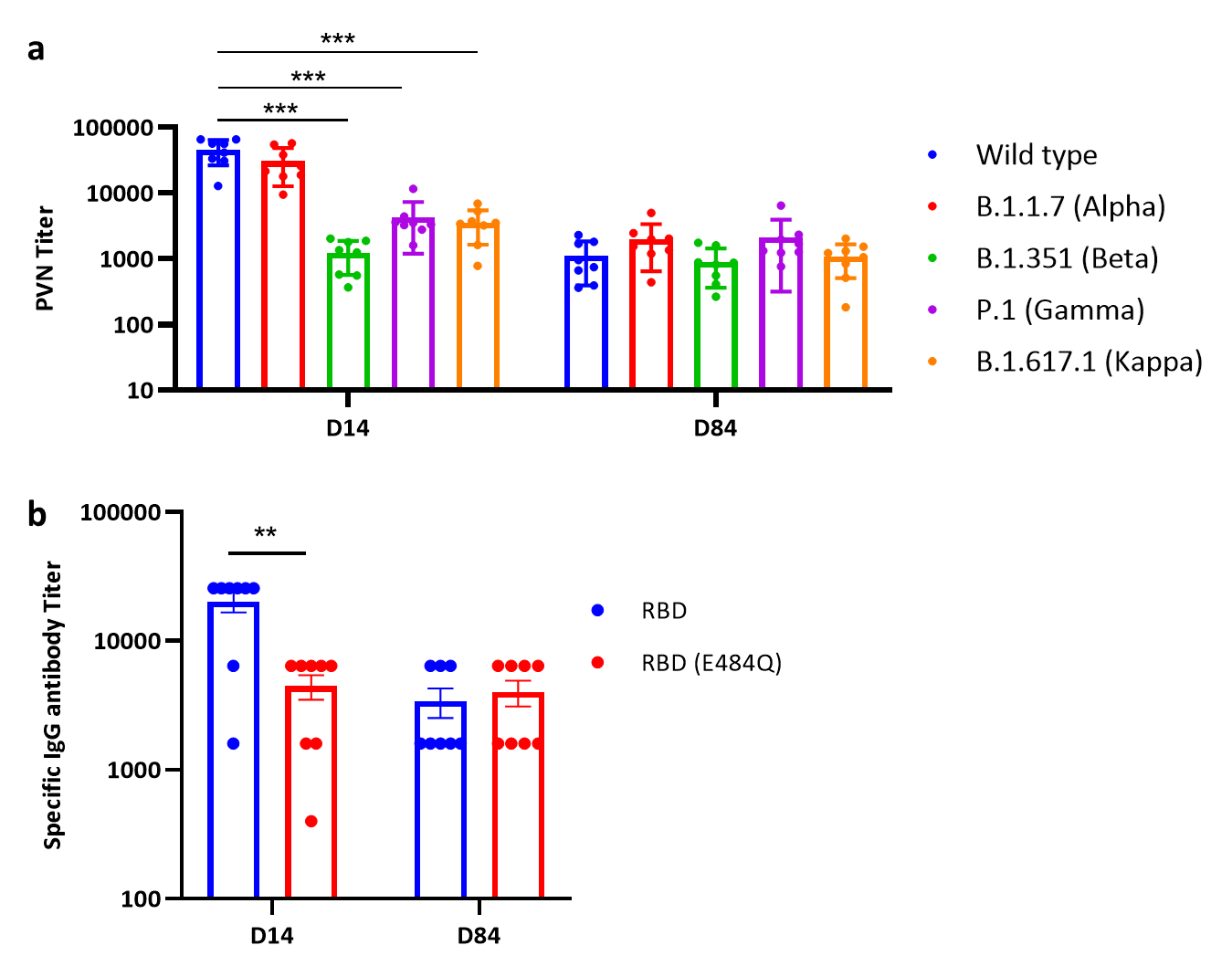


**Figure S3. Pseudovirus Neutralizing antibodies and Anti-RBD IgG antibodies induced by S-trimer adjuvanted by PIKA in rabbits.** Rabbits were vaccinated intramuscularly twice (Days 0 and 7) with the S-trimer Vaccine adjuvanted by PIKA. Serum were obtained at day 14 and 84 after initial immunization for pseudovirus neutralization assays and anti-IgG ELISA assay.

(a) Neutralizing ability of serum against Wuhan-1 (blue), B.1.1.7 (red), and B.1.351 (green), P.1 (purple) and B.1.617 (orange) mutants.

(b) Anti-IgG ELISA assay measure binding activity of wild-type and E484Q RBDs.

Points represent individual animals. Data are shown as mean ± SEM. Statistical significance was determined using a one-way ANOVA with multiple comparisons tests (**p* < 0.05, ***p* < 0.001, ****p* < 0.001).


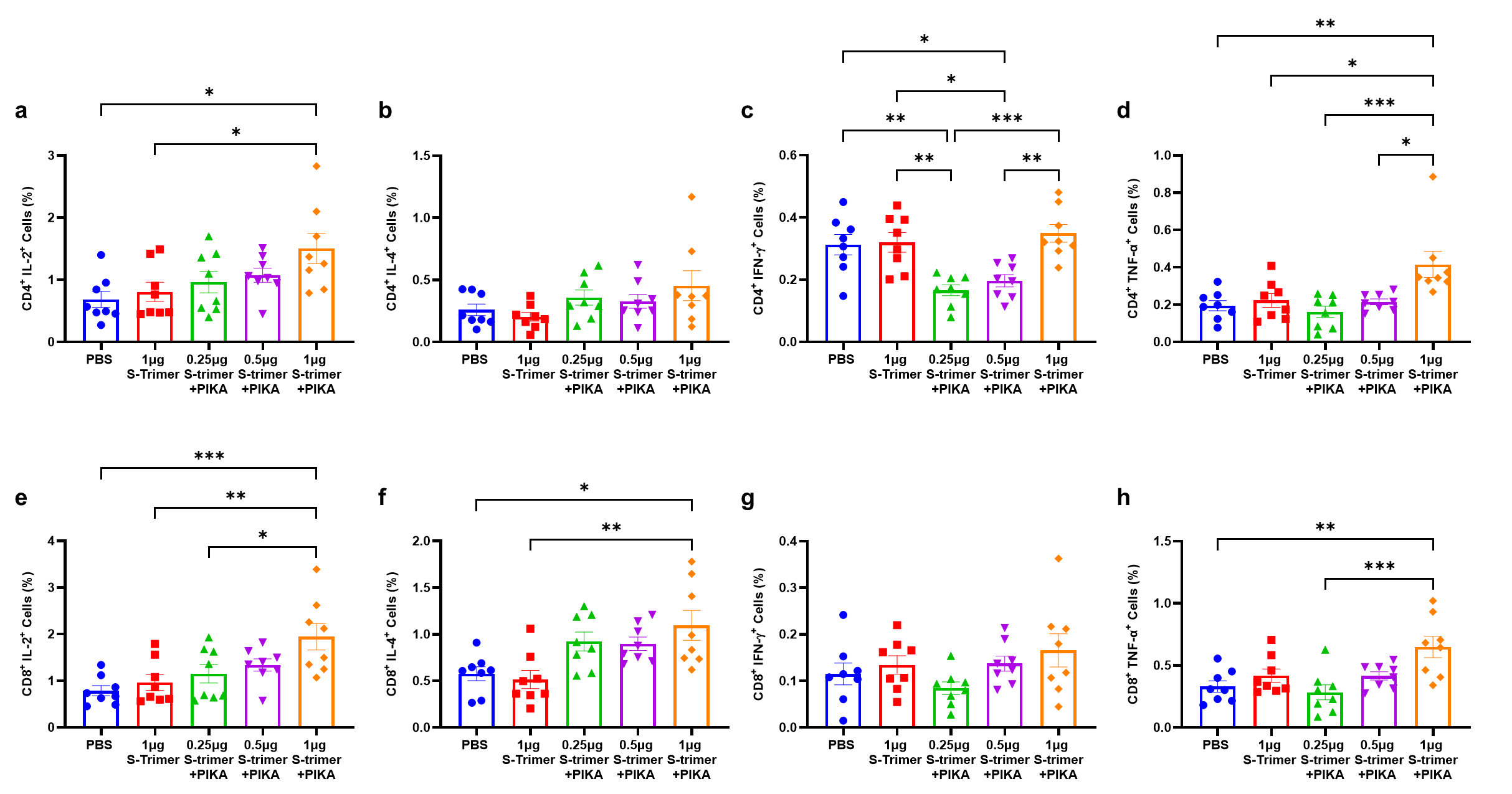


**Figure S4. SARS-CoV-2-Specific T Cell Immune Response of PIKA Adjuvanted S-trimer in Mice.** BALB/c mice (n=8/group) were immunized with various doses of S-Trimer with or without PIKA adjuvant three times on Day 0, Day 7 and Day 14. Splenocytes were harvested from mice at 21 days after the first immunization and stimulated with the S-trimer protein (4 μg/ml), followed by detection of intracellular cytokine staining by flow cytometry.

(a-d) The proportion of CD4+ T cells producing IL-2, IL-4, IFN-γ and TNF-α among all CD4+ T cells were evaluated by intracellular cytokine staining.

(e-h) The proportion of CD8+ T cells producing IL-2, IL-4, IFN-γ and TNF-α among all CD8+ T cells were evaluated by intracellular cytokine staining.

Data are shown as mean ± SEM. Significance was calculated using a one-way ANOVA with multiple comparisons tests (**p* < 0.05, ***p* < 0.01, ****p* < 0.001, *****p* < 0.0001).

**Supplementary Table 1: Lung pathology scores post infection.**

| Group | Animal  ID | Left upper lung | | Left middle lung | | Left Low lung | | Right upper lung | | Right middle lung | | Right Low lung | | Right accessory lung | | Total  Sccore |
| --- | --- | --- | --- | --- | --- | --- | --- | --- | --- | --- | --- | --- | --- | --- | --- | --- |
|  |  | Lesion  Area | Inflammation  Degree | Lesion  Area | Inflammation  Degree | Lesion  Area | Inflammation  Degree | Lesion  Area | Inflammation  Degree | Lesion  Area | Inflammation  Degree | Lesion  Area | Inflammation  Degree | Lesion  Area | Inflammation  Degree |  |
| 5μg S-trimer+PIKA  (0-7) | 16139 | 0 | 0 | 1 | 1 | 2 | 1 | 0 | 0 | 2 | 1 | 1 | 1 | 2 | 1 | 13 |
|  | 16143 | 2 | 1 | 3 | 3 | 2 | 2 | 0 | 0 | 2 | 1 | 2 | 1 | 2 | 1 | 22 |
|  | 16109 | 0 | 0 | 0 | 0 | 1 | 1 | 0 | 0 | 0 | 0 | 0 | 0 | 0 | 0 | 2 |
|  | 16149 | 0 | 0 | 0 | 0 | 1 | 1 | 1 | 1 | 2 | 1 | 2 | 1 | 0 | 0 | 10 |
| 20μg S-trimer+PIKA  (0-7) | 16151 | 1 | 1 | 2 | 2 | 1 | 1 | 1 | 1 | 0 | 0 | 1 | 1 | 1 | 1 | 14 |
|  | 15139 | 2 | 1 | 2 | 1 | 2 | 1 | 2 | 1 | 3 | 1 | 3 | 2 | 3 | 1 | 25 |
|  | 16153 | 0 | 0 | 2 | 1 | 0 | 0 | 0 | 0 | 1 | 1 | 1 | 1 | 1 | 1 | 9 |
|  | 16137 | 1 | 1 | 3 | 1 | 0 | 0 | 0 | 0 | 2 | 1 | 0 | 0 | 2 | 1 | 12 |
| 20μg S-trimer+PIKA  (0-7-14) | 16129 | 2 | 1 | 2 | 1 | 1 | 1 | 2 | 1 | 1 | 1 | 3 | 3 | 1 | 1 | 21 |
|  | 16161 | 0 | 0 | 1 | 1 | 1 | 1 | 0 | 0 | 0 | 0 | 1 | 1 | 1 | 1 | 8 |
|  | 12103 | 0 | 0 | 0 | 0 | 2 | 1 | 2 | 1 | 1 | 1 | 1 | 1 | 1 | 1 | 12 |
|  | 15121 | 1 | 1 | 1 | 1 | 2 | 1 | 2 | 2 | 2 | 1 | 0 | 0 | 2 | 2 | 18 |
| PBS | 16117 | 2 | 2 | 2 | 2 | 3 | 2 | 2 | 2 | 3 | 2 | 2 | 2 | 3 | 2 | 31 |
|  | 16155 | 3 | 2 | 1 | 1 | 2 | 2 | 2 | 3 | 1 | 1 | 3 | 2 | 3 | 3 | 29 |
|  | 14117 | 1 | 1 | 2 | 1 | 3 | 2 | 3 | 1 | 3 | 2 | 2 | 2 | 3 | 3 | 29 |
|  | 14113 | 0 | 0 | 2 | 2 | 3 | 3 | 1 | 1 | 2 | 2 | 2 | 2 | 2 | 1 | 23 |
